# Long-term cardio-vascular risk assessment in chronic kidney disease and kidney transplanted patients following SARS-COV-2 disease: protocol for multi-center observational match controlled trial

**DOI:** 10.1186/s12882-022-02809-4

**Published:** 2022-05-06

**Authors:** Laura Tapoi, Mugurel Apetrii, Gianina Dodi, Ionut Nistor, Luminita Voroneanu, Lucian Siriteanu, Mihai Onofriescu, Mehmet Kanbay, Adrian Covic

**Affiliations:** 1 Department of Internal Medicine, Nephrology and Geriatrics, Grigore T Popa University of Medicine and Pharmacy, Iasi, Romania; 2 Cardiovascular Diseases Institute Prof. Dr. George I.M. Georgescu, Iasi, Romania; 3 Department of Nephrology, Dr C I Parhon University Hospital, Iasi, Romania; 4 Department of Medicine, Division of Nephrology, Koc University School of Medicine, Istanbul, Turkey

**Keywords:** SARS-CoV-2, Cardiovascular disease, Cardio-vascular risk, Chronic kidney disease, dialysis, Kidney transplant

## Abstract

**Background:**

The coronavirus disease (COVID-19) caused by the severe acute respiratory syndrome coronavirus 2 (SARS-CoV-2) produced a pandemic since March 2020 by affecting more than 243 million people with more than 5 million deaths globally. SARS-CoV-2 infection is produced by binding to angiotensin-converting enzyme, which among other sites is highly expressed in the endothelial cells of the blood vessels, pericytes and the heart, as well as in renal podocytes and proximal tubular epithelial cells. SARS-CoV-2 and cardiovascular disease (CVD) are interconnected by risk factors association with an increased incidence of the disease and by determining de novo cardiac complications. At the same time, COVID-19 disease can lead to acute kidney injury directly, or due to sepsis, multi-organ failure and shock. Therefore, the pre-existence of both CVD and chronic kidney disease (CKD) is linked with a higher risk of severe disease and worse prognosis.

**Methods:**

The main aim of this study is to assess the CV risk in a CKD (stage 3 to 5), dialysis and kidney transplanted population, following SARS-CoV-2 infection, with focus on the endothelial dysfunction as compared to a control group of matched patients. By using clinical evaluation, flow-mediated dilatation, carotid-femoral pulse wave velocity, intima-media thickness, echocardiographic parameters, lung ultrasound, bioimpedance spectroscopy and a series of novel biomarkers, the investigators will determine the long-term impact of this disease on CV and renal outcomes.

**Discussion:**

This study will address the challenges and implications in long-term CV sequeale of COVID-19 and focus on a better understanding of the underlying mechanisms and possible therapeutic options.

**Trial registration:**

Patient enrolment in the trial started in January 2021 and is expected to finish at the end of 2022. The study can be found on ClinicalTrials.gov database with NCT05125913 identifier.

Registered on 18 November 2021 - Retrospectively registered.

## Background

The coronavirus disease (COVID-19) caused by the severe acute respiratory syndrome coronavirus 2 (SARS-CoV-2) first emerged in early December 2019 and was declared a pandemic on March 2020 [1]. Since then, the SARS-CoV-2 continues to infect millions of individuals worldwide, with more than 243 million confirmed cases and up to 5 million deaths [2]. With nearly 35,707 new detected cases in the last 24 hours (at the time of writing, 26th of October 2021), as declared by World Health Organization (WHO), the SARS-CoV-2 pandemic revives, producing a cascade of diagnostic and therapeutic challenges.

SARS-CoV-2 infection is produced by binding to angiotensin-converting enzyme (ACE2), which among other sites is highly expressed in the endothelial cells of the blood vessels, pericytes and the heart, as well as in renal podocytes and proximal tubular epithelial cells. Of note, ACE2 RNA expression in the kidney is nearly 100-fold higher than that in the lungs [3]. Autopsy studies detected the presence of SARS-CoV-2 in both myocardium [4] and renal tissue [5], suggesting that COVID-19 profoundly influences the cardiovascular (CV) system and the kidneys and this may lead to long-termed cardio-pulmonary-renal consequences.

According to the COVID-19 global literature platform available on WHO website, there are 388,238 documents that cover more than 60 well-known scientific databases, such as Medline, Scopus, Web of Science, PubMed, Science Direct, etc. Nearly 845 studies were found using “cardiovascular” and “renal/kidney” keywords, which describe the effects on both systems among SARS-CoV-2 patients, even if it is acknowledged that clinical course of severe COVID-19 can impair rapidly from mild to critical with renal and heart diseases. Among these, we will mention an observational retrospective cohort study, published by Naser et al. [6] that evaluates the risk factors, predictions, and progression of acute kidney injury (AKI) in 353 hospitalized COVID-19 male patients from the BDF, Royal Medical Services in Bahrain. The detailed analysis of laboratory kidney function testing revealed that 168 patients (47.6%) developed different AKI stages. Furthermore, the AKI group showed elevated levels of inflammatory, cardiac injury, and fibrinolysis markers at time of admission, when compared with the non-AKI group. According to the author’s data upon admission, the potential predictors of AKI in COVID-19 are age, the frequencies of the comorbidity, diabetes, hypertension, chronic kidney disease (CKD), cardiovascular disease (CVD) and the severity of pneumonia. When analysing the recovery versus death rate for 87 deaths (51.8%) were recorded for AKI development in comparison to only 4 deaths (2.2%) for non-AKI group. It is important to remark that the authors did not make any suggestions regarding causal relationships between exposure and AKI, just recommending that clinicians should perform kidney function tests on admission and follow-up in hospitalized COVID-19 patients. In the Brazilian multi-center cohort study recently published, one in every five kidney transplant (KTx) patients died after being diagnosed with COVID-19, and the rate of death was significantly higher in those with AKI, mainly when renal replacement therapy was vital [7]. The study included 1680 KTx patients diagnosed with SARS-CoV-2 virus at 5,9 years after transplantation, from 35 Brazilian centres, from which 88.6% reported comorbidities, mainly hypertension, diabetes and CVD. In the call to action publication of the ERA-EDTA working group, CKD is defined as the most prevalent comorbidity for severe COVID-19, along with dialysis and organ transplantation, therefore several urgent action should be taken to prevent severe COVID-19 for this group of patients [8].

Another case report published by Chibane et al. [9] this year highlights that COVID-19 is associated with hyperacute multi-organ thromboembolic storm, existent at a 66-year old female patient admitted for pre-syncope, with post-mortem diagnosis of the infection. Transthoracic echocardiography evidenced pulmonary thromboembolism that was followed by an ischemic stroke and death, despite anticoagulation therapy. Therefore, the concept of coronavirus infection of endothelial cells and hypercoagulability could be supported, even in the absence of respiratory symptoms.

In June 2021, Rao et al. [10] published a large, multicenter, retrospective study that associates AKI with mortality in patients hospitalized with COVID-19, and demonstrates for the first time the relationship between AKI and CV events in COVID-19 for the hospitalization period. The American Heart Association’s (AHA) COVID-19 CVD registry enrolled 8574 patients with COVID-19 and CKD, end-stage kidney disease (ESKD) and AKI. According to multivariate adjustment, CKD and ESKD were not associated with mortality or major adverse cardiac events in the acute episode of SARS-CoV-2 infection, an unexpected finding given prior studies linking CKD with higher mortality in COVID-19 [11]. The authors explained the difference by the complexity of the data collection and multivariate models with extensive tuning for covariates.

As expected, COVID-19, the kidneys and the CV system are linked in a bidirectional way. On one hand, both CVD and renal disease represent a risk amplifier for morbi-mortality in the COVID-19 setting [12, 13]; on the other hand COVID-19 can exacerbate associated CVD and determine de novo cardiac complications (acute myocardial injury, stress cardiomyopathy, myocarditis, pericarditis, arrhythmias, heart failure and cardiogenic shock) [3, 12]. Furthermore, myocardial injury is also an important prognostic factor for the disease severity [14], as well as in-hospital and long-term mortality [14, 15]. At the same time, COVID-19 can lead to AKI directly, or due to sepsis, multi-organ failure and shock [3, 16].

More and more studies are reporting the persistence of COVID-19 related symptoms for more than a month after the acute episode, recently defined as post-COVID syndrome*.* The percentage of incessant symptomatology varies from 35 to 89.2% [17, 18] and it is independently associated with age [17], two or more comorbidities and the severity of the acute disease [18–21]. It looks that women are more predisposed to experience remaining symptoms [22]. The laboratory abnormalities are also frequently reported. An increase in D-dimer, interleukin-6 (IL-6), C-reactive protein (CRP), fibrinogen level as well as lymphocyte count may serve as predictors for long COVID [23]. The percentage of renal transplant recipients who survived acute COVID-19 and had no clinical symptoms or were free from any laboratory abnormalities after 2 months from the initial diagnosis was 11.53%. Sustained clinical symptoms were found in 45.2%, and one or more laboratory abnormalities were reported in 71.2%. Long-term complications in these patients were strongly predicted by diabetes mellitus, hospitalization in the acute setting, renal allograft function, as well as laboratory abnormalities [24].

COVID-19 survivors have higher risks of AKI, de novo development or worsening of proteinuria, eGFR decline and progression to ESKD. These outcomes are reported even among patients those with mild symptoms and increase with the severity of the infection [25].

The underlying mechanisms of these manifestations and long-term complications are not completely understood, but emerging data emphasize the endothelium, as a central pillar in the long COVID conundrum, with implications in inflammation, the pro-thrombotic phenotype and disseminated intravascular coagulation [26]. Endothelial dysfunction (ED) refers to a systemic condition in which the endothelium loses its physiological properties, including the tendency to promote vasodilation, fibrinolysis, and anti-aggregation [13]. Morphologic findings from deceased patients confirm the presence of viral elements within endothelial cells and an accumulation of inflammatory cells, with evidence of endothelial and inflammatory cell death. Endotheliitis in several organs as a direct consequence of viral involvement and of the host inflammatory response may explain the impaired microcirculatory function in different vascular beds and clinical sequelae in patients with COVID-19 [27].

In this context, the European Society of Cardiology recently stated the need for further research to elucidate the role of endothelium in COVID-19 and to assess the long-term CV effects following COVID-19 [26].

## Methods

**Fig. 1:**
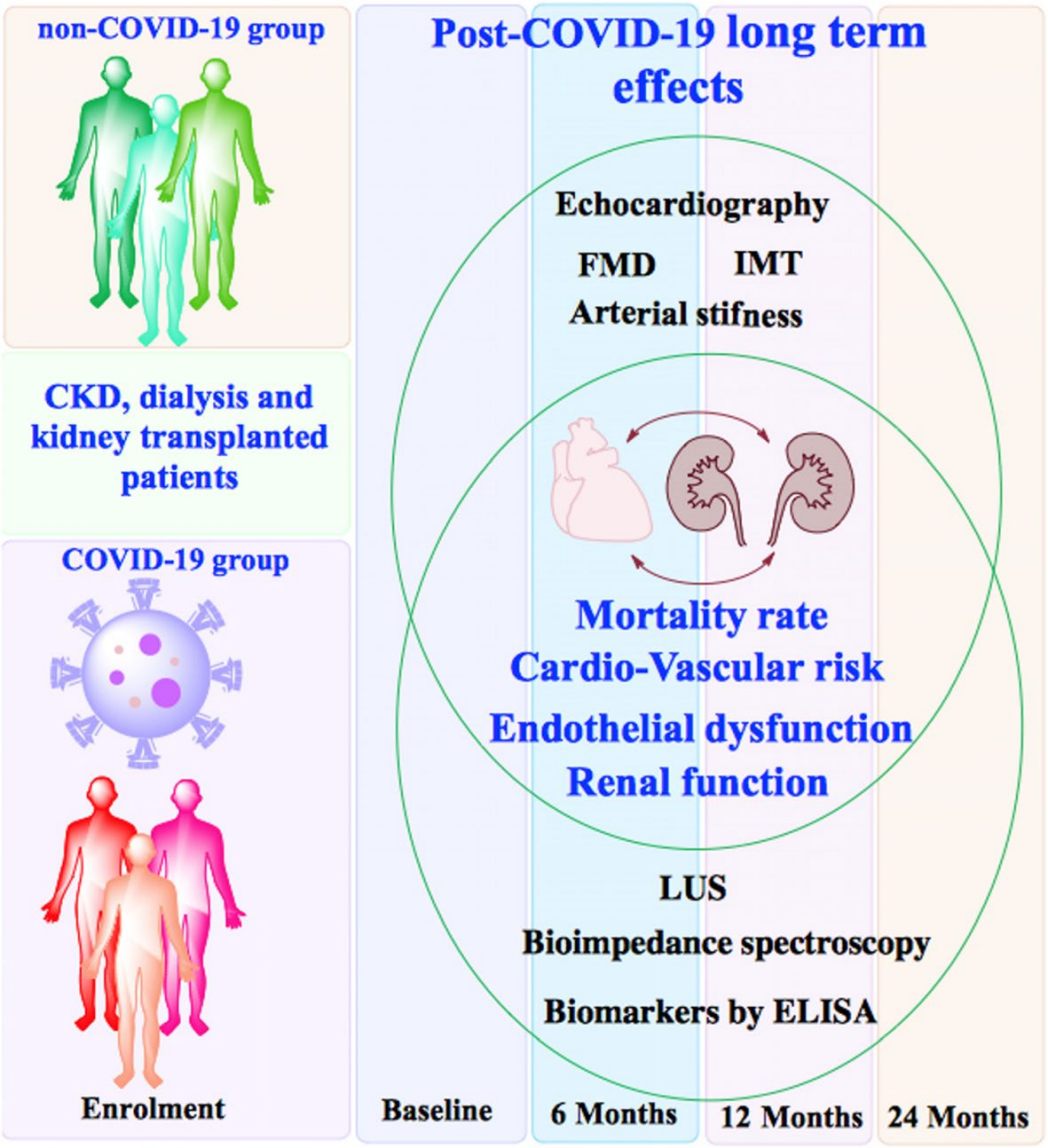
Protocol flowchart

Data emerging from the general population suggests that COVID-19 is essentially an endothelial disease, with possible deleterious long-term effects that are currently incompletely understood. Therefore, our aim is to assess the CV risk in a CKD, dialysis and KTx population, following SARS-CoV-2 infection, by determining the long-term impact of this disease on CV and renal outcomes in the aforementioned population as compared to a control group of matched patients (see Fig. 1).

### Objectives

The objectives of this observational study are to determine:

- the feasibility of flow mediated dilation (FMD), bioimpedance spectroscopy (BIS) fluid assessment, lung ultrasound (LUS) and pulse wave velocity (PWV) measurement in CKD patients following COVID-19 and matched CKD control-patients without a history of previous SARS-CoV-2 infection;
- the long-term impact of the COVID-19 on markers of CV risk and ED in patients with CKD and KTx on a composite CV outcome (time to first non-fatal myocardial infarction, non-fatal stroke, hospitalization for heart failure or CV death) and on all-cause mortality;
- the long-term impact of the COVID-19 on markers of CV risk and ED in patients with CKD and KTx on a composite renal outcome: doubling of creatinine or a 40% decline in eGFR or dialysis initiation.

### Study design

This observational cohort study will include CKD (stage 3 to 5), dialysis and KTx patients from dialysis unit’s auxiliary to Dr. C.I. Parhon University Hospital, Iasi, Romania, diagnosed with COVID-19 according to the interim guidelines of the WHO [28] using the reverse transcriptase polymerase chain reaction (RT-PCR) assay from nasal or throat swab. Patients from North-East of Romania with a documented history of SARS-CoV-2 will be recruited into the CARDIO SCARS IN CKD observational cohort study. All participants must have recovered from acute illness due to SARS-CoV-2 virus infection.

### Study population

We aim to enrol 250 patients, divided into two groups with minimum 125 patients per group: COVID group and non-COVID group. Both groups will be monitored at 6, 12 and 24 months since the first procedure visit, defined as the baseline.

#### Inclusion criteria

- Age > 18 years;
- Patients with CKD stage 3–5, including dialysis or KTx with confirmed COVID-19, at minimum 2 weeks after the confirmed test;
- Age, sex and kidney disease (CKD stage 3–5, dialysis or KTx) matched patients without confirmed SARS-CoV-2 infection.

#### Exclusion criteria

- Prior diagnosis of pulmonary fibrosis, pneumectomy or massive pleural effusion;
- Active malignancies;
- Pregnancy;
- Active systemic infections (due to difficulties in the interpretation of nonspecific inflammation biomarkers in this type of patients);
- Congenital heart disease.

### Approval

All procedures performed in this study were in accordance with the ethics standards of the institutional and/or national research committee and with the 1964 Helsinki declaration and its later amendments. This study was approved by the Ethical Committee of Grigore *T. Popa* University of Medicine and Pharmacy of Iasi (no. 110/2021). The enrolled patients will receive a written informed consent approved by the Ethical Committee, that describes the nature of the trial, aims and expected advantages, as well as possible risks, and all study participants will provide signed consent prior to enrolment.

### Screening and enrolment

**Fig. 2:**
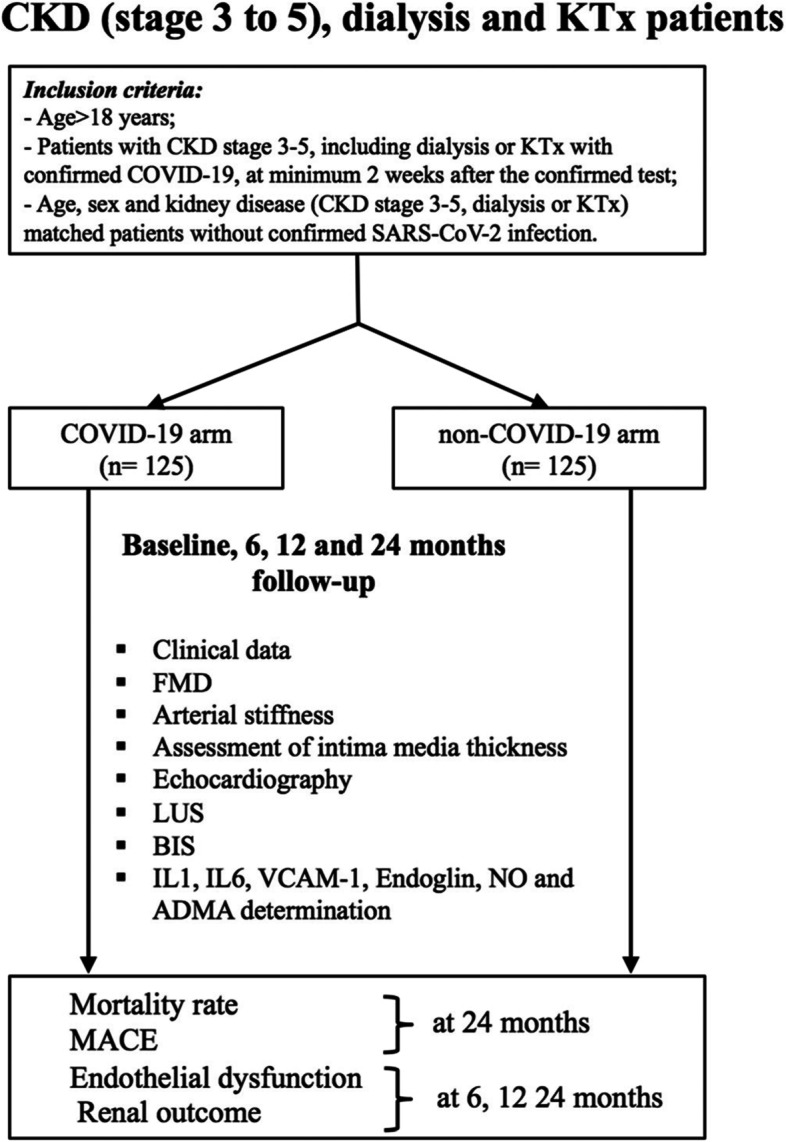
Flow diagram of the study design

All study procedures (as pointed in Table 1 and Fig. 2), including screening and enrolment, will be conducted by trained personnel with backgrounds in cardiology, nephrology, internal medicine and medical science. We will recruit participants from the investigators’ clinic patient panel at Dr. CI Parhon Clinical Hospital of Iasi and dialysis units from Iasi. Investigators will also partner with other providers from nearby hospitals and/or dialysis centres for enrolment. We will approach participants during routine clinic visits. For retention, we will leverage the electronic medical record to track hospitalizations and scheduled clinic visits during the study period. All participants will first undergo a brief screening discussion, via telephone or in person, to determine eligibility and interest. Participants will be queried on their health history including the presence of other major medical illnesses, to ensure that no exclusion criteria are present. If eligible, participants will be invited to participate in a 1-hour baseline assessment, when each participant will undertake a detailed interview in order to summarize the patient datasheet, further included into the trial database. During the baseline assessment, informed consent will be presented by the study staff including a description and timing of the study procedures, potential risks and benefits of study involvement, rights to withdraw from the study, and details regarding protections against study risks. All participants will receive a copy of their signed informed consent form.

**Table 1:** Study procedures flowchart

Procedures/Timepoint		Study interventions			
	Enrolment	Baseline	6 months	12 months	24 months
**Enrolment**					
*Eligibility screen*	*X*				
*Contact information*	*X*				
*Informed consent*	*X*				
**Interventions**					
*FMD*		*X*	*X*	*X*	*X*
*Arterial stiffness*		*X*	*X*	*X*	*X*
*Assessment of intima media thickness*		*X*	*X*	*X*	*X*
*Echocardiography*		*X*	*X*	*X*	*X*
*LUS*		*X*	*X*	*X*	*X*
*BIS*		*X*	*X*	*X*	*X*
*Blood collection*		*X*	*X*	*X*	*X*
**Assessments**					
*Clinical data*	*X*	*X*	*X*	*X*	*X*
*IL1, IL6, VCAM-1, endoglin, NO and ADMA*		*X*	*X*	*X*	*X*
*CV risk*			*X*	*X*	*X*
*Mortality rate*					*X*
*Renal outcome*			*X*	*X*	*X*
*ED*					*X*

After the consent process, patients will undergo the study interventions as detailed below. At the completion of the baseline intervention, the patients will be scheduled for their second intervention session approximately 6 months later. Participants will be informed that they will continue to receive their usual medical care. After each intervention visit, the patients will receive a copy of their records, data that will be available to their physician if needed.

### Study interventions

All the proposed performed procedures are described in Table 2 with a special attention on the used protocol and the obtained result.

**Table 2:** Protocol for the study procedures

Procedure	Protocol description
FMD	- use of Philips CX50 Ultrasound System with a 12-Mhz probe; - the vasoactive medications removal for 24 h before the procedure; - patient in supine position for at least 15 min before the examination; - the right arm will be immobilized in the extended position for the brachial artery 2–4 cm above the antecubital fossa recording; the contralateral arm will be used if an arteriovenous fistula is present; - 3 adjacent measurements of end-diastolic brachial artery diameter will be made from single 2D frames; - the cuff will be inflated up to 50 mmHg higher value of systolic blood pressure and kept this way for 5 minutes; - the maximum FMD diameters will be analysed from the average of the 3 consecutive maximum diameter measurements, and 1 minute after the cuff will be deflated; - the FMD will be calculated as the percentage change in diameter compared with baseline resting diameters, in agreement with the criteria set by the International Brachial Artery Reactivity Task Force.
Arterial stiffness	- applanation tonometry using SphygmoCorTM; PWV Inc., Westmead, Sydney, Australia; - the patient will be recumbent 10 minutes before the measures; - measurements: sequentially pulse of the carotid and femoral, the transit time from the R-wave of the simultaneously acquired electrocardiogram to the foot of the carotid and femoral pulse, and the difference acquired electrocardiogram to the foot of the carotid and femoral pulse; - the carotid-femoral PWV will be calculated from the difference between these 2 transit times divided by distances measured from the body surface (arterial path length).
IMT	- Philips CX50 Ultrasound System with a 12 MHz probe; - high-resolution B-mode ultrasound of the common carotid arteries with scanning of the longitudinal axis until the bifurcation and of the transversal axis will be performed; - two longitudinal measurements will be obtained for each carotid artery, by rotating the vessels at 180^o^ increments along their axis; - IMT will be measured at 1 cm proximal to the bifurcation on each side.
Echocardiography	- according to the American Society of Echocardiography recommendations by an observer unaware of the LUS and bioimpedance results; - measurements: cardiac anatomy (e.g. volumes, geometry, mass) and function (e.g. left ventricular function, valvular function, right ventricular function, and pulmonary artery pressure); - in patients with adequate acoustic windows, both left ventricular global longitudinal strain (LVGLS) and right ventricular free wall strain (RVFWS) will be calculated.
LUS	- patient in the supine position; - measurements of the anterior and lateral chest on both sides of the chest, from the second to the fourth (on the right side to the fifth) intercostal spaces, at parasternal to mid-axillary lines; - B-lines will be recorded in each intercostal space and were defined as a hyperechoic, coherent US bundle at narrow basis going from the transducer to the limit of the screen; − B-lines starting from the pleural line can be either localized or scattered to the whole lung and be present as isolated or multiple artifacts; - the sum of B-lines produces a score reflecting the extent of lung water accumulation (0 being no detectable B-line).
BIS	- portable whole-body multi-frequency bioimpedance analysis device (BCM®Body Composition Monitor – Fresenius Medical Care D GmbH); - electrodes will be attached to the patient’s forearm and ipsilateral ankle, with the patient in a supine position; - measurements: body resistance and reactance to electrical currents of 50 discrete frequencies, ranging between 5 and 1000 kHz; - calculations: ECW, ICW and TBW, to determine the amount of fluid overload; - AFO is defined as the difference between the expected patient’s ECW under normal physiological conditions and the actual ECW, whereas RFO is defined as the absolute fluid overload AFO to ECW ratio.
Blood collection for biomarker evaluation	- whole blood will be collected in gel & clot activator tubes (minimum 3.5 mL tube) at each intervention visit for each patient; - serum will be collected by centrifugation and frozen in aliquots at −80 ° C until further analysis; - IL1, IL6, VCAM-1, endoglin, NO and ADMA in serum samples will analysed by specific enzyme linked immunosorbent assay (ELISA) kits on a Sunrise Basic Tecan microplate reader.

### Data collection and follow-up

Data will be collected on paper-based forms and in a secure electronic database for further analysis.

#### Clinical data

Personal data (age, sex, height); CV risk factors (smoking, alcohol use, weight, body mass index); comorbidities (diabetes, hypertension, coronary artery disease, heart failure etc); physical examination (blood pressure, heart rate, crackles, pedal edema etc); type of renal disease; renal function (urea, creatinine, proteinuria, etc); medication; inflammation evaluation: CRP; complete blood count will be collected.

For the dialysis population: dialysis vintage and for the transplantation population: dialysis vintage before transplantation; date of transplantation, type (living or cadaveric donor), mismatch number, type of induction therapy; type of chronic immunosuppression therapy, will be also collected. Supplementary, for the COVID arm, we will assess also the severity of the infection, administered treatment, use of oxygen therapy, vaccination status.

### Outcome data

***The primary outcomes of this study*** will be all-cause mortality rate, a composite CV outcome (time to first non-fatal myocardial infarction, non-fatal stroke, and hospitalization for heart failure or CV death) and long-term impact of the COVID-19 on markers of CV risk and ED in all included patients.

***Secondary outcomes*** are defined as a composite of renal outcome: doubling of creatinine or a 40% decline in eGFR or dialysis initiation in CKD or KTx patients.

### Strategies to ensure adequate enrolment and protocol compliance

Report forms will be uploaded into a secure database in a timely manner. This will allow periodical checks for enrolment rates, data accuracy and protocol compliance.

### Statistical analysis

The statistical analyses will be performed using the SPSS for Windows, version 19.0.1, R (version 3.1.2) package for statistical analysis and STATA 13. Non-normally distributed variables will be expressed as median with interquartile range and normally distributed variables as mean ± SD, as appropriate. Between-group comparisons will be assessed for nominal variables with the Chi-square test, and by Kruskal-Wallis test or one-way ANOVA for the rest of the variables. In order to comply with the assumptions for regression analysis, logarithmic conversion will be performed for non-normally distributed variables. Pearson correlation coefficient will be used to determine correlations between variables. Stepwise multivariate regression analysis including all univariate associates (*p* < 0.05) will be used to assess the independent associations between variables. Survival and time-to-event analysis of outcomes will be performed using Kaplan-Meier cumulative survival plots and Cox proportional hazards model, including adjustment for potential confounding factors. In the multivariate Cox models, we will adjust for all variables that will be correlated to the study outcomes with *P* < 0.05 at univariate Cox analyses.

### Sample size power calculation

According to sample size power calculations, including 125 patients in each group will provide statistical power (alpha 0.05 and power 80%) for an estimated difference in the mortality rate of 9% [29] in the non-COVID group compared to 22% mortality in the COVID positive group [30–34].

### Strengths and limitations of the study protocol

To our best knowledge, this is the first study that aims to evaluate the long-term CV risk after COVID-19 disease in CKD, dialysis or KTx patients. By using a holistic approach, this study can contribute to a better understanding of the long-COVID syndrome and can improve the risk stratification in a special population that already associates an increased CV risk.

Due to nature of the study, i.e. clinical evaluation of CKD stage 3–5, dialysis or KTx patients with a confirmed positive result for SARS-CoV-2 virus, and the lack of previous experimental work on this particular virus, we can encounter several limitations, briefly described below:

- patient’s refusal to continue with the trial;
- the impossibility of performing speckle-tracking echocardiography in all patients due to suboptimal images (poor acoustic window) or to metallic joint prostheses, cardiac stent or pacemakers, decompensated cirrhosis and limb amputation patients, due to bioimpedance technique limitations;
- the difficulty for the arterial stiffness assessment in obese patients.

## Discussion

Recent studies show a growing interest in long-term CV sequeale of COVID-19 and focus on a better understanding of the underlying mechanisms and possible therapeutic options.

Analysing the active clinical trials registered at Clinicaltrials.gov, regarding the post-COVID-19 syndrome manifestations, there are several found studies as presented in Table 3, at both CV and renal level, but none dealing with the purpose of this study.

**Table 3:** Active clinical trials concerning to post- COVID-19 syndrome

Condition	Outcomes	Type	Trial identifier
COVID-19	Long-term morbidities and sequels of SARS-CoV-2 infections in the general population (NAPKON-POP)	Observational on 2000 participants (estimated)	NCT04679584, recruiting
COVID-19, Cardiac Disease	Sequelae (organ dysfunction) after COVID-19 (12 months)	Observational on 120 participants (estimated)	NCT04442789, recruiting
COVID-19, Hypertension	Average 24 hour Ambulatory Blood Pressure Monitoring - Systolic Blood Pressure, (all day and night) at 12 months in SARS-CoV-2 cases	Observational on 150 participants (estimated)	NCT05087290, recruiting
COVID-19	Native myocardial T1 relaxation time (MRI) at 12 weeks post COVID-19 diagnosis	Observational on 215 participants (actual enrolment)	NCT04525404, active, not recruiting
COVID-19	Presence of at least one clinical, biological and/or imaging cardiovascular anomaly within 1 month of recovering	Observational on 200 participants (estimated)	NCT04452630, recruiting
COVID-19	Incidence of major cardiovascular events (congestive heart failure, myocardial infarction, cardiomyopathy and ischemic stroke) and of atrial arrhythmia, 12 months post-COVID-19	Observational (Patient Registry) on 100 participants (estimated)	NCT04605965, recruiting
COVID-19	Echocardiographic strain measurements of the left, right heart and vascular ultrasound findings up to 12 months	Observational on 250 participants (estimated)	NCT04756193, recruiting
COVID-19, ARDS	Evaluation of evolution of renal and right and left myocardic functions during first year after ICU discharged in population studied	Observational on 150 participants (estimated)	NCT04401111, recruiting
COVID-19, AKI	GFR loss at 6 months post-hospital admission, Cystatin C as indicator of mortality, respiratory illness and disease severity at 30 days post-hospital admission	Observational (Patient Registry) on 900 participants (estimated)	NCT04353583, recruiting
COVID-19, Kidney Transplant	Predictive value of IL-6 contents of whole blood samples after ex vivo stimulation with LPS and ATP over 10 months	Interventional (Clinical Trial) on 115 participants (estimated)	NCT04369456, recruiting
COVID-19, AKI, CKD, ESRD, Transplant, Failure, Kidney	AKI incidence from hospital admission through hospital discharge up to 24 weeks, dialysis requirement through study completion up to 1 year from enrolment, hospital mortality within 1 year, renal functional recovery assessed at 3, 6 and 12 months from enrolment at hospital admission	Observational on 2000 participants (estimated)	NCT04491227, Enrolling by invitation

The comprehensive review of Nalbandian et al. [21] describes based on available current literature on post-acute COVID-19 syndrome, the most persistent symptoms and/or long-term complications beyond 4 weeks from the start SARS-CoV-2 replication. Briefly, besides, the usual fatigue, decline in quality of life, muscular weakness and pain, the authors put together an organ-specific complications of post-acute COVID-19. At CV level, the authors included palpitations, dyspnea and chest pain as symptoms, along with increased cardiometabolic demand, myocardial fibrosis or scarring, arrhythmias, tachycardia and autonomic dysfunction. At renal level, reduced eGFR has been reported at 6 months follow-up and persistent impaired renal function.

MRI studies indicate that myocardial inflammation persists more than 2 months after the resolution of the acute episode [35] and myocardial injury can occur even in previously healthy subjects with mild or asymptomatic forms of COVID-19 [36]. Myocardial inflammation can lead to fibrotic remodelling which in turn manifests through alteration of cardiac relaxation. In 48 patients that were evaluated by transthoracic echocardiography at 6 months distance from COVID-19, myocardial injury identified by elevations in cardiac troponin during the acute phase was significantly associated with diastolic dysfunction, but this abnormality was revealed only after mild exercise. Systolic left ventricle and right ventricular functions were normal, and there were no significant valvulopathies or pericardial disease [37].

Compared with controls, in patients with COVID-19, both left ventricular (ejection fraction, GLS) and right ventricular systolic function (TAPSE, RVLS) are significantly reduced when compared to controls and independently associated with higher mortality rates [38–41]. RVFWS is altered even in mild forms of COVID-19 and not necessarily in the presence of biomarkers indicating myocardial injury. However, RVFWS is more impaired in those with biochemical evidence of myocardial injury and is directly correlated with the disease severity [42]. Studies regarding the prognostic role of right ventricular fractional area change (RVFAC) have contradictory results, as this parameter is inconsistently associated with mortality rate [42]. Right ventricular diameter was also correlated with mortality rates [40, 43]. Most studies suggest that both right ventricular and left ventricular function improve at follow-up [42, 44], although there is also data that indicate that left ventricular function can remain impaired [44]. IL-6 levels could predict right ventricular systolic dysfunction in patients with COVID-19 [45].

LUS is a widely used investigation that can describe lung parenchymal abnormalities by identifying B-lines, B-line like artefacts, consolidation or pleural line irregularities. The presence of B-lines was significantly correlated with disease severity and longer duration of symptoms, as well as with increased mortality [46]. Moreover, LUS has practical application in documenting pulmonary sequelae in patients with severe COVID-19 pneumonia and can predict its progression [47].

FMD is a widely recognized indicator of both subclinical atherosclerosis and coronary artery endothelial function [48], with important prognostic value [49]. Compared with healthy controls, significantly lower FMD values were reported during the acute phase of COVID-19 [50, 51]. In addition, FMD was an independent predictor of mortality in this setting [51, 52]. FMD was also assessed in the post-COVID population, with evidence suggesting the persistence of impaired FMD at 3 months distance after the acute event [53]. In a study that included 133 convalescent moderate-to-severe COVID-19 patients and 133 matched controls, FMD values were significantly lower within 2 months from COVID-19, and were directly correlated with the severity of pulmonary disease. However, this difference was nullified by female gender, smoking or the presence of more than 3 concomitant risk factors [54]. Thus, one can conclude that COVID-19 is an independent predictor of ED [55]. Interestingly, pulmonary rehabilitation increased FMD with ~ 2% among 82 patients with a history of severe or critical COVID-19 [56].

Arterial stiffness is a risk factor for in-hospital mortality in COVID-19 patients [57] and also for increased hospitalization duration [58]. PWV is also increased in patients with prior COVID-19 disease [59, 60].

The up-to-date review of Samprathi and Jayashree [61] presents the rational use of available biomarkers in the diagnosis and management of patients with COVID-19. The authors discuss based on Sars-CoV-2 pathophysiology, the laboratory tests offered to the clinician, from hematological, inflammatory, coagulative and biochemical parameters and up to cardiac biomarkers. The combined hematological biomarkers, namely increased leukocytes, neutrophils, CRP, PCT and ferritin, cytokine levels (IL-2R, IL-6, IL-8, IL-10 and TNF-α) and decreased lymphocyte counts predicts the severity of the disease and hospital admission. At inflammatory level, elevated CRP, PCT, cytokines (IL-6, IL-1b, IL-2, IL-8, IL-17, G-CSF, GMCSF, IP-10, MCP-1, CCL3, and TNFα) values forecast also, predicts progression to severe disease. Raised cardiac biomarkers involving CK, CK-MB, LDH, Mb, cTnI, α-HBDH, AST, and NT-proBNP were observed especially in severe illness and were associated with higher mortality. The proposed biomarkers play an essential role in early suspicion, diagnosis, monitoring, and recognition of acute complications, but what about on the long-term effects of COVID-19?

In July 2021, Andrianto et al. [62] conducted a literature search for observational studies that analysed the relationship between ED biomarkers and clinical outcomes in COVID-19 patients. In a meta-analysis that included 1187 patients from 17 studies, VWF antigen, PAI-1, t-PA, sTM were predictors of worse outcome in COVID-19 patients. The elevated biomarkers that display an endothelial dysfunction in COVID-19 increase also the risk of vascular complications, poor outcomes, and death.

Overall, we can disclose that since COVID-19 is not a simple upper respiratory tract disease, and more a multisystemic one that affects many organs, it generates long-term permanent sequelae with a serious burden on individuals and the health systems in the near future.

## Data Availability

The datasets used and/or analysed during the current study are available from the corresponding author on reasonable request, namely Dr. Gianina Dodi at gianina.dodi@umfiasi.ro.

## Abbreviations

ACE-2: Angiotensin-converting enzyme 2
ADMA: Asymmetric dimethylarginine
AFO: Absolute fluid overload
AHA: American Heart Association
α-HBDH: Alpha-hydroxybutyrate dehydrogenase
AKI: Acute kidney injury
AST: Aspartate aminotransferase
BIS: Bioimpedance spectroscopy
CCL3: Macrophage inflammatory protein-1 α
CK: Creatine kinase
CKD: Chronic kidney disease
CK-MB: Creatine kinase-myocardial bound
COVID-19: Coronavirus disease 2019
CRP: C-reactive protein
cTnI: Cardiac troponin I
CV: Cardiovascular
CVD: Cardiovascular disease
ECW: Extracellular water
ED: Endothelial dysfunction
eGFR: Estimated Glomerular Filtration Rate
ELISA: Enzyme linked immunosorbent assay
ERA-EDTA: European Renal Association- European Dialysis and Transplant Association
ESKD: End stage kidney disease
FMD: Flow-mediated dilation
G-CSF: Granulocyte colony-stimulating factor
GMCSF: Granulocyte-macrophage colony-stimulating factor
ICAM-1: Intercellular Adhesion Molecule 1
ICW: Intracellular water
IL-: Interleukin-
IP-10: Interferon gamma-induced protein 10
KTx: Kidney transplant
LDH: Lactate dehydrogenase
LUS: Lung ultrasonography
LVGLS: Left ventricular global longitudinal strain
Mb: Myoglobin
MCP-1: Monocyte chemoattractant protein-1
MRI: Magnetic resonance imaging
NO: Nitric oxide
NT-proBNP: N-terminal pro b-type natriuretic peptide
PAI-1: Plasminogen activator inhibitor-1 antigen
PCT: Procalcitonin
PWV: Pulse wave velocity
RFO: Relative fluid overload
RNA: Ribonucleic acid
RT-PCR: Reverse transcriptase polymerase chain reaction
RVFWS: Right ventricular free wall strain
RVFAC: Right ventricular fractional area change
RVLS: Right ventricular systolic function
SARS-CoV-2: Severe acute respiratory syndrome coronavirus-2
sTM: Soluble thrombomodulin
TAPSE: Tricuspid annular plane systolic excursion
TBW: Total body water
TNF-α: Tumor necrosis factor α
t-PA: Tissue-type plasminogen activator
VCAM-1: Vascular cell adhesion molecule 1
VWF: von Willebrand factor
WHO: World Health Organization
